# Aspects of Regulation of Xenotransplantation in Europe

**DOI:** 10.3389/ti.2024.13349

**Published:** 2024-12-13

**Authors:** Ralf Reinhard Tönjes

**Affiliations:** Paul-Ehrlich-Institut, Federal Institute for Vaccines and Biomedicines, Langen, Germany

**Keywords:** regulation, Council of Europe, European Union, xenotransplantation, advanced therapy medicinal products

## Abstract

The Council of Europe (CoE) and the European Union (EU) share the same fundamental values, i.e., human rights, democracy and the rule of law, but are separate entities which perform different, yet complementary, roles. The CoE brings together governments from across Europe, and beyond, to agree minimum legal standards in a wide range of areas. CoE monitors how countries apply the standards that they have chosen to sign up to. It provides technical assistance, often working together with the EU. The EU refers to those same European values as a key element of its political and economic integration processes. It often builds upon CoE standards when drawing up legal instruments and agreements which apply to the member states, furthermore, monitoring work in its dealings with neighbouring countries, many of which are CoE member states. At CoE, the European Committee on Organ Transplantation (CD-P-TO) is the steering committee in charge of organ transplantation activities. In the EU, the regulation on Substances of Human Origin (SoHO) was endorsed in 2024. The CoE and the EU thave concluded an agreement expanding their co-operation in the field of SoHO. In the BTC regulation, xenotransplantation is not included.

## Council of Europe

Founded in 1949, the CoE has 46 member states. The CoE is an international organization with the aim to protect human rights, democracy as well as the rule of law in all member states. The CoE cannot make binding laws, however it enforces international agreements reached by European states on various topics to achieve recommendations, conventions and treaties (https://www.coe.int/en/web/portal/european-union).

In 1999, the Committee of Ministers of the CoE set up a working party on XTx under the joint responsibility of the Steering Committee on Bioethics (CDBI) and the European Health Committee (CDSP) which decided to prepare a report on the state of the art in the field of XTx. The Report as well as an Explanatory Report to Recommendation Rec (2003)10 of the Committee of Ministers to member states on xenotranplantation were published in 2003 [1–3]. The report proposed that member states establish a mechanism for the registration and regulation of “certain aspects of XTx including (a) basic research and clinical trials, (b) the source and care of animals for use in XTx, (c) XTx programmes, (d) long term follow-up and review of XTx recipients and (e) the XTx source animals” [1]. The Recommendation Rec (2003)10 took “into account the shortage of organs and tissues of human origin available for transplantation” and it considered that “XTx might be one of the possible therapeutic responses to this shortage,” further “noting that XTx remains largely an experimental activity and that research is essential for the achievement of progress in this field.” Moreover, it stated the awareness “of the risks of rejection and illness XTx may cause in the recipient patient.” Eventually, the recommendation was “mindful of the considerable risks which might arise from XTx in the field of public health and the transmission of diseases” [3].

The topic of XTx encompassing definitions and infectious risk is described briefly in the “Guide to the quality and safety of tissues and cells for human application” (T&C Guide); Chapter 5. Donor Evaluation, Subchapter 5.34 Relative Contraindications, f. Xenotransplantation; CD-P-TO, EDQM, 5th Edition, 2022 [4]. In the T&C Guide, XTx is defined as “any procedure that involves the transplantation, implantation or infusion into a human recipient of either (a) live cells, tissues, or organs from a nonhuman animal source, or (b) human body fluids, cells, tissues or organs that have had *ex vivo* contact with live nonhuman animal cells, tissues or organs. Biological products, drugs, or medical devices sourced from non-human animals that do not contain living cells, tissues or organs, including (but not limited to) porcine insulin, porcine heart valves, porcine skin and acellular porcine corneal stroma, and collagen matrices derived from acellular porcine, bovine or any other xenogeneic source are not considered as xenotransplantation” [4].

In 2023, the European Directorate for the Quality of Medicines & Healthcare (EDQM), European Committee (Partial Agreement) on Organ Transplantation (CD-P-TO) at the CoE accepted a proposal of member states to launch an update of the given report. The update was suggested due to the recent progress achieved in non-clinical (preclinical) and clinical activities in pig organ (heart, kidney) xenotransplantation to humans, particularly the xenotransplantations of genetically modified (gm) pig hearts to patients, performed as individual medical treatments in 2022 and 2023, Baltimore, United States, as well as recent activities in tissue and cellular xenotransplantation and extracorporeal exposure to xenoorgans, xenotissues and xenocells.

The state of the art of immunological and physiological barriers, non-clinical activities and of gm pigs as animal donors should be updated. Moreover, cultural, ethical and religious aspects of xenotransplantation, national policies as well as legislative and regulatory frameworks should be highlighted.

The updated report will not be placed neither as a legal instrument nor as a guide for transplant professionals, but rather as an overview of progress in the biomedicinal field which moves closer to the clinical arena. However, the update of the report may provide a basis for national legislation, European legislation as well as for international coordination and cooperation.

The timetable, designed for the years 2024–2026, is based on the formation of a working group formed by experts of the member states, the assignment of topics and chapters, drafting of an updated report and the consultation of the final report. At current, the working group elaborates an update of a questionnaire which was addressed to the member states in 1999 to compile the existing documents, laws and regulations within the CoE.

In view of the dynamics in the field of xenotransplantation, the World Health Organisation (WHO) that is part of the initiative suggested to implement other, non-European countries and societies in the project, such as The Transplantation Society (TTS) and the International Xenotransplantation Association (IXA).

## European Union

In the European Commission Directive 2006/17/EC (§ 1.1.13 of Annex I) “transplantation with xenografts” is included as general criteria for exclusion of deceased human donors of tissues and cells, related to the fact that exposure of human recipients to non-human live animal material has the potential for cross-species infection, caused by infectious agents such as porcine endogenous retroviruses (PERV) that are integrated into the pig genome [5, 6] and other exogenous infectious agents, e.g., porcine cytomegalovirus (PCMV) [7].

The EU comprises 27 member states. In the EU, a regulatory framework for XTx is based on guidelines and ordinances on advanced therapy medicinal products (ATMP) [8], pharmacovigilance and clinical trials.

The fundamental rights of both animals as donors and humans as recipients of organs, tissues, and cells are adequately protected by the framework.

Moreover, in the EU member states, national laws may be implemented, such as those on genetic engineering, protection against infection and medicinal products, e.g., the German Medicinal Products Act.

The following definitions shall apply for the purposes of the ATMP regulation (EC) No 1394/2007 [8]:

“(a) ‘Advanced therapy medicinal product’ means any of the following medicinal products for human use: (1) a gene therapy medicinal product as defined in Part IV of Annex I to Directive 2001/83/EC, (2) a somatic cell therapy medicinal product as defined in Part IV of Annex I to Directive 2001/83/EC, (3) a tissue engineered product as defined in point (b).

(b) ‘tissue engineered product’ means a product that (1) contains or consists of engineered cells or tissues, and (2) is presented as having properties for, or is used in or administered to human beings with a view to regenerating, repairing or replacing a human tissue.

A tissue engineered product may contain cells or tissues of human or animal origin, or both. The cells or tissues may be viable or non-viable. It may also contain additional substances, such as cellular products, bio-molecules, bio-materials, chemical substances, scaffolds or matrices.

Products containing or consisting exclusively of non-viable human or animal cells and/or tissues, which do not contain any viable cells or tissues and which do not act principally by pharmacological, immunological or metabolic action, shall be excluded from this definition.

(c) Cells or tissues shall be considered ‘engineered’ if they fulfil at least one of the following conditions: the cells or tissues have been subject to substantial manipulation, so that biological characteristics, physiological functions or structural properties relevant for the intended regeneration, repair or replacement are achieved. The manipulations listed in Annex I, in particular, shall not be considered as substantial manipulations.”

For XTx, the ATMP regulation displays some limitations since animal organs are not explicitly mentioned, even though they may be derived from genetically modified (gm) animals. As a consequence, those animal organs would be substantially manipulated compared with organs that are harvested from wild-type animals.

In the ATMP regulation, the definition of somatic cell therapeutics, as well as the definition of tissue-engineered products of animal origin, is based on tissues or cells; however, it excludes organs. Naturally, organs derived from gm animals contain tissues and cells.

To this end, the European Medicines Agency (EMA, Amsterdam, Netherlands) has published the guideline on xenogeneic cell-based medicinal products [9]. “This guideline addresses the scientific requirements for xenogeneic cell-based medicinal products for human use. Xenogeneic cell-based medicinal products contain viable animal cells or tissues as the active substance. Xenogeneic materials might be sourced either from non-transgenic or transgenic animals. The animal cells can also be genetically modified. Although not within the scope of the guidance, some of general principles of this guideline will apply to viable animal cells used as raw materials (e.g., feeder cells) and/or where contamination with xenogeneic material is possible. This guideline is intended for products entering the marketing authorisation (MA) procedure. However, the principles laid down in the guideline should be considered by applicants entering into clinical trials.” The legal basis for the guideline should be read in conjunction with ATMP regulation (EC) No 1394/2007 [8].

Central elements of the ATMP regulation include (a) designation of the EMA to grant marketing authorizations for XTx products within the EU, (b) requirement for traceability of xenogeneic organs, tissues and cells, from creation through clinical use and ultimate disposition, and (c) hospital exemption for medicinal products that are not routinely prepared.

In the EU, regulatory pathways to yield marketing authorizations for medicinal products, including ATMP, are based on data that cover product quality, nonclinical assessment (i.e., preclinical trials), as well as clinical trials.

Data must be summarized by the applicant, often the pharmaceutical entrepreneur working in partnership with clinical investigators and their medical institution(s), in dossiers including an internationally standardized set of Common Technical Documents (CTD). The application is evaluated by the European National Competent Authorities (NCA; in Germany the Paul-Ehrlich-Institut, Federal Institute for Vaccines and Biomedicines, Langen) that are nominated as rapporteur and co-rapporteur by EMA. The CTD are expected to show consistent data on the quality, safety, and efficacy of the particular product.

Beforehand, EMA and NCA offer central and national scientific recommendations on the classification of ATMP.

To ensure that research, development, and regulation remain current, the regulatory framework in the EU and its member states will be adjusted to appropiately reflect scientific and technical advancements in xenotransplantation.

In the EU, the regulation on Substances of Human Origin (SoHO) covering blood, tissues and cells (BTC), except solid organs, was endorsed in 2024. The CoE/EDQM and the EU, through the European Commission, have concluded an agreement expanding the scope of their co-operation in the field of SoHO^1^. The field of XTx is not part of the BTC regulation.

## World Health Organisation

The WHO has a long-standing interest in xenotransplantation, which started with the publication of the “WHO Guidance on Xenogeneic Infection/Disease Surveillance and Response: A strategy for International Cooperation and Coordination” in 2001 [10].

The next step was resolution WHA57.18 of the 57th World Health Assembly in 2004, urging member states, amongst others, to perform xenogeneic transplantation only “when effective national regulatory control and surveillance mechanisms overseen by national health authorities are in place” [11].

This resolution was preceded by a position report on ethical aspects published by the IXA pointing to the requirements of adequate preclinical data, proper oversight by competent authorities, and approval by institutional bodies over-seeing the ethical conduct of human research and animal welfare [12].

Resolution 57.18 led to four WHO-supported global consultations. The first Xenotransplantation Advisory Consultation was held in Geneva, Switzerland, in 2005 [13].

It was followed by the First WHO Global Consultation on Regulatory Requirements for Xenotransplantation Clinical Trials, which was organized in collaboration with the Ministry of Health of China, the International Xenotransplantation Association and the University of Central-South China in Changsha, China, in 2008. The recommendations were published as the “Changsha Communiqué” [14] to develop and to update regulatory requirements for xenotransplantation clinical trials.

The Second WHO Global Consultation on Regulatory Requirements for Xenotransplantation Clinical Trials was organized in collaboration with the TTS and the IXA in Geneva, Switzerland, in 2011, and had a focus on xenotransplantation-associated infectious risk [15, 16].

The Third WHO Global Consultation on Regulatory Requirements for Xenotransplantation Clinical Trials was organized in collaboration with the IXA and the EMA in Changsha, China, December 2018 [17]. The principles and recommendations of the “Changsha Communiqué” were reviewed and discussed in detail by different working parties charged with covering the following topics: (a) xenozoonosis; (b) regulatory; (c) biorepository; (d) transgenic pig facilities; (e) biomaterials and encapsulation; and (f) immunosuppression and tolerance induction. The guidance document from the Second Global Consultation (Geneva, 2011) was included in these discussions.

Eventually, the consultation focused on drafting proposed revisions of the WHO documents, and resulted in the formulation of the draft “Third WHO Global Consultation on Regulatory Requirements for Xenotransplantation Clinical Trials, The 2018 Changsha Communiqué” [17].

The draft was submitted to WHO in February 2019 for WHO and World Health Assembly consideration where it has not been finalized yet.

## Conclusion

XTx is separately and partially covered by the CoE, the EU, as well as the World Health Organisation (WHO). So far, there is indirect interaction and partial linkage between the COE, the EU and the WHO.

## Footnotes

1
https://www.edqm.eu/en/-/the-council-of-europe/edqm-and-the-european-union-conclude-an-agreement-expanding-the-scope-of-their-co-operation-in-the-field-of-substances-of-human-origin
